# Smart city construction and urban green development: empirical evidence from China

**DOI:** 10.1038/s41598-023-44061-2

**Published:** 2023-10-13

**Authors:** Youzhi Zhang, Yinke Liu, Jing Zhao, Jingyi Wang

**Affiliations:** 1 School of Economics Management, Xi’an Shiyou University, Xi’an, 710065 Shaanxi China; 2 School of Economics Management, Xi’an University of Technology, Xi’an, 710054 Shaanxi China

**Keywords:** Ecology, Environmental social sciences

## Abstract

Smart city construction represents an advanced stage of China's urbanisation process and plays an important role in promoting green economic growth and sustainable development. Propensity score matching is combined with the difference-in-difference method to analyse the data of 221 prefecture-level cities in China from 2006 to 2020 to assess the impact of smart city construction on urban green development. We found that smart city construction can significantly contribute to urban green development; this contribution has long-term benefits. Further analysis shows that smart city construction promotes urban green development via industrial structure and green technology innovation and that smart city construction has a significant positive spatial spillover effect, i.e., it promotes urban green development locally while significantly contributing to urban green development in neighbouring regions.

## Introduction

Since the reform and opening up, the quality of China's urban development has rapidly improved, but the various urban diseases that accompany this development cannot be ignored. Furthermore, while environmental pollution has become an increasingly serious problem, urban green development has been severely constrained. By the end of 2022, China's urbanisation level, driven by large-scale industrialisation, reached 65.22%, creating rapid economic growth while causing the urban ecology to severely deteriorate and environmental problems to increase; this traditional, unsophisticated urban development model needs to be changed^1^. The current trend for innovative urban development models addresses the deteriorating ecological environment. As an advanced stage of China's new urbanisation, smart city construction is characterised by innovation-driven and environmentally sustainable development. "Smart" refers to a new urban development mode featuring intelligence, digitalisation and informatisation. The core of smart cities involves using big data, the Internet of Things, cloud computing and other information technologies to promote city development via digitalisation and intelligence and improve city operation efficiency^2^. To define smart cities, Yigitcanlar et al. state that smart cities represent a new concept in which technological progress and urban development are integrated; furthermore, they act as advanced systems that are socially driven, technologically driven, and policy driven^3^. Lom et al. categorise the concept of the smart city as an information-physical system, one of whose main objectives is to enable collaboration between sectoral systems and the systems to which they belong, thus improving the quality of urban life, saving energy and reducing carbon emissions^4^.

## Literature review

Since the US company IBM put forwards the idea of a “Smart Earth” in 2008, countries worldwide have been gradually promoting the construction of intelligent cities. As early as 2012, China’s Ministry of Housing and Urban‒Rural Development officially began piloting smart cities and gradually expanded the scope of the pilot in 2013 and 2014^5–7^. Most of the current academic research on smart city construction focuses on the concept and implementation of smart city construction; in assessing the effectiveness of smart city construction, economic benefits are mainly determined from the input‒output perspective, with few studies focusing on the concurrent social and ecological benefits. We executed an empirical investigation regarding the impact of smart city construction on urban green development and its mechanism of action. Therefore, the relevant studies on smart cities are sorted out and grouped into three categories: economic effect, innovation effect and environmental effect. In terms of economic effects, some studies have shown that smart city construction can significantly improve the quality of urban development, adjust the industrial structure and promote sustainable economic development^8–10^. Jo et al. studied the impact of smart city construction on the industrial ecosystem in Korea and found that smart industries are becoming pillar industries that create new value chains; furthermore, smart city construction is energising smart industries and promoting the transformation and upgrading of industrial structures^11^. Liu et al. noted that smart city construction plays a significant role in promoting high-quality economic development in China^12^. Furthermore, Zhao et al. empirically tested the mechanism and long-term dynamic effects of smart city construction on China's high-quality economic development, thus showing that the impact of smart city construction on China's high-quality economic development has increased over time^13^. In terms of innovation effects, Caragliu et al. used European patent data as a measurement of the level of technological innovation and found that smart city construction accelerates urban innovation; this effect is more pronounced in areas of high-tech patents^14^. Chu et al. empirically tested the relationship between smart city construction and urban innovativeness in China and pointed out that smart city construction mainly enhances urban innovativeness through smart government^15^. For the environmental effect, with the increasingly severe problem of "big city disease", the connotation of smart cities has gradually evolved into a new urban development mode that emphasises green and sustainable development. Liu et al. noted that smart city construction can significantly contribute to green economic growth and is regionally heterogeneous^16^. Shen et al. and Wu et al. treated smart city construction as a quasinatural experiment, and their empirical results showed that smart city construction contributed significantly to environmental pollution^17,18^.

At present, most of the research on green development in academia focuses on horizontal measurement and impact factors. The first method of measuring green development is to establish an input‒output indicator system and use the data envelope analysis (DEA) method to measure total factor productivity to characterise urban green development^19–21^. The second is to adopt a comprehensive indicator evaluation system, using the entropy method to measure comprehensive indicators to characterise urban green development^22–24^. In terms of the analysis of influencing factors, Qiu et al. showed that low-carbon city pilot policies are important for achieving environmentally friendly economic transformation and are conducive to urban green development^25^. Zhang used a spatial econometric approach to analyse the impact of technological innovation on urban green development and found that green technological innovation that enhances urban eco-efficiency is more conducive to urban green development in China's eastern region; furthermore, efforts to promote this kind of development were received favourably at the administrative level of the city^26^. Current academic research regarding the impact of smart city construction on urban green development is relatively scarce, but in-depth promotion of green smart city construction is an inevitable trend for future urban construction and development^27^. Du et al. measured the level of urban green development with the GML index and used the panel data of 172 cities in China from 2007 to 2017 to empirically analyse the impact of smart city construction on urban green development efficiency; their results showed that smart city construction has a positive effect on urban green development efficiency^28^. Zhang et al. used the SBM-DEA model to measure the efficiency of urban green innovation and analysed the impact of constructing smart cities; they obtained similar conclusions^29^.

In view of the abovementioned information, this paper makes the following marginal contributions. First, using panel data of 221 prefecture-level cities from 2006 to 2020, the study scope and time are expanded, and a combination of the difference-in-difference (DID) method and propensity score matching (PSM) is used to overcome sample selection bias. Second, a mediating effect analysis is conducted to explore the important channels through which smart city construction influences urban green development. Third, a spatial Durbin double difference model is developed to control for spatial dependence, which allows for a spatial spillover effects analysis of the impact of pilot smart city construction areas on urban green development in neighbouring nonpilot area cities.

## Theoretical analysis and research hypothesis

Information technology and big data network platforms, which are intrinsic to smart cities, link the various economic agents in the city and fully emphasise the intelligence of governance, the convenience of residents' lives and the digitalisation of enterprise production. In terms of government governance, smart city construction promotes intelligent governance that can more effectively promote environmental regulation and energy management, improve the structure of energy consumption and promote the use of clean energy. In terms of residential life, smart cities provide residents with smart homes that reduce energy consumption by intelligently regulating indoor temperature, lighting and electricity consumption. In addition, smart cities improve the transport system and reduce traffic congestion, thereby reducing fuel consumption and carbon emissions. In terms of enterprise production, enterprises can invest in factors of production more efficiently through industrial IoT and big data analysis, expanding the stage of increasing returns to scale and thus reducing the waste of resources. On the other hand, smart city construction helps enterprises carry out supply chain management more effectively and use digital tools to conduct environmental impact assessments, thus promoting the transition to green and sustainable development. The above factors are conducive to high-quality economic and sustainable resource development and promote green urban development. We further classify the theoretical mechanisms of smart city construction for urban green development into industrial structure effects, technological innovation effects and policy spillover effects.

The industrial structure effect is an important channel through which smart city construction influences urban green development. Many scholars have noted that industrial structure upgrading is the basis for economic growth. This upgrading leads to a reduction in environmental pollution and an increase in production efficiency, thus promoting green total factor productivity^30^. Furthermore, smart city construction can use information technology and technological innovation to rationalise, and thus upgrade, industrial structures. In addition, smart city construction generates new technology that can impact traditional industries; new industries act in a "demonstration role", forcing traditional industries to adjust and upgrade. For example, traditional high pollution and high energy consumption enterprises can use modern information technology to adjust their industrial structure and adapt to the market, thus allowing them to move towards high technology, low energy consumption and low pollution. Therefore, the industrial structure of the city can be transformed and upgraded, thus promoting green economic development.

The effect of technological innovation is an important driving force that causes smart city construction to influence urban green development. To promote smart city construction, the government provides financial and policy support for technological innovation. It is willing to invest in multiple research and development resources to accelerate the Internet of Things, cloud computing and big data and promote the innovative development of a new generation of information technology. However, technological innovation will in turn stimulate the level of green economic development in cities^31^. In the framework of Schumpeter's theory, enterprises are the main body of innovation. To adapt to city development trends and gain competitive advantages, enterprises will seize development opportunities, maximise information technology and continuously promote technological innovation. These actions will reduce the cost and improve the ability of enterprises to treat pollution, which will benefit the development of the green economy. In addition, the government encourages green production and living and low-carbon consumption under smart city construction, which subtly forms and strengthens people's awareness of environmental protection. These actions generate new demand for green products and give rise to various new production processes and technologies while reducing the amount of resources consumed, improving energy use efficiency, providing new growth drivers for economic development and helping to promote urban green development.

**Figure 1:**
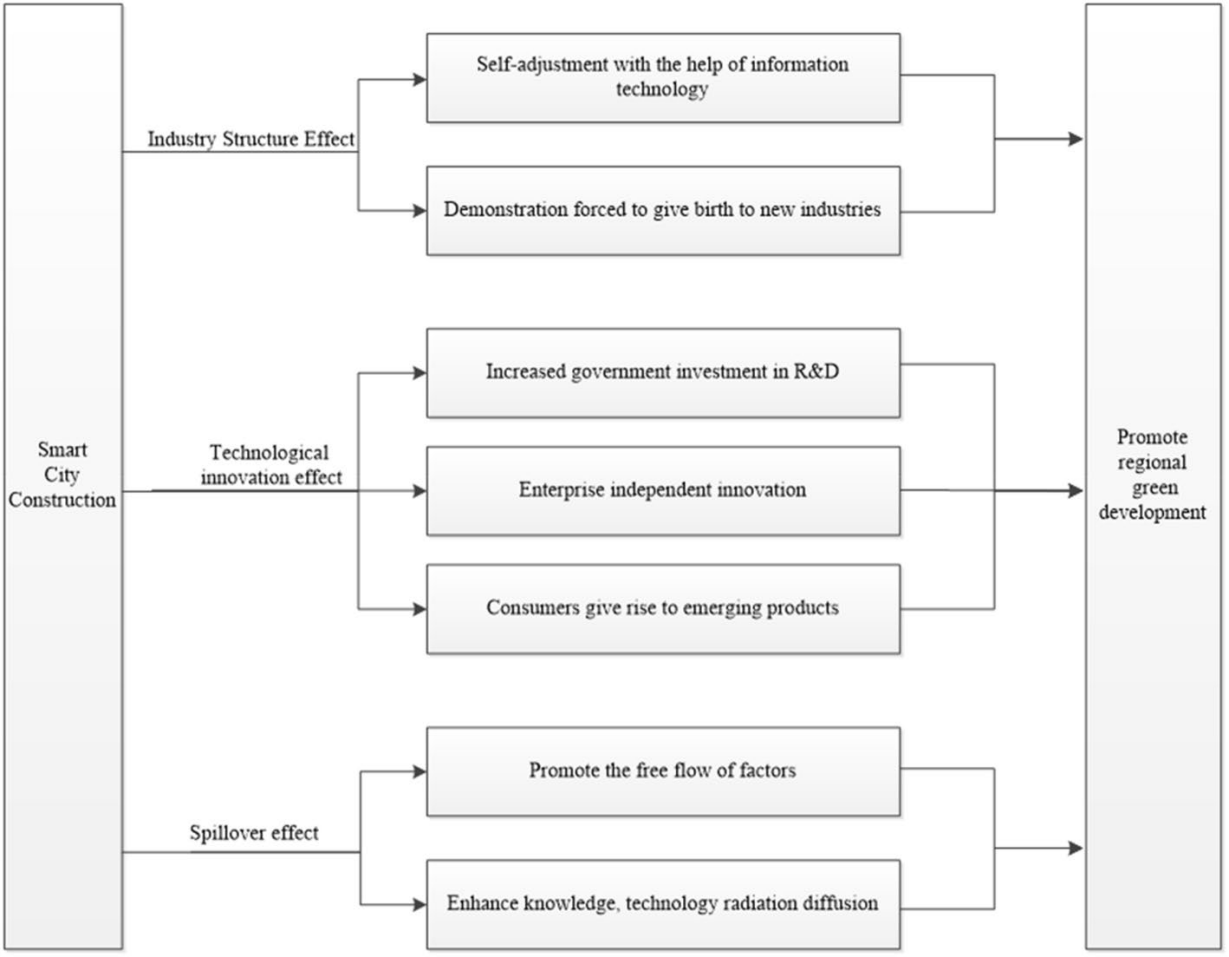
Theoretical analysis.

From the perspective of economic geography, the implementation of smart city construction in the region will promote urban green development in other neighbouring regions. On the one hand, smart city construction originates from the Internet of Things, cloud computing and other big data network platform support. Information technology and other intelligent elements are not restricted by time and space, which can break the barriers to factor flow and is conducive to promoting cobusiness, cobuilding and sharing between regions and strengthening economic ties. On the other hand, the construction of smart cities can optimise the internal systems of cities, such as the financial development environment, education level and infrastructure construction in the pilot areas, continuously attracting the inflow of various resource elements. These elements then give rise to the clustering of new industries such as smart industries and modern service industries led by new business models, which in turn drive urban green development in neighbouring cities through knowledge, technology and other spillover radiation (Fig. 1).

Based on the abovementioned information, we propose the following hypotheses:

- Hypothesis 1: Smart city construction plays a significant role in promoting urban green development.
- Hypothesis 2: Smart city construction promotes urban green development through industrial structure and green technology innovation.
- Hypothesis 3: Smart city construction has a positive spatial spillover effect on urban green development in neighbouring areas.

## Research design

### Variable selection

Dependent variable: Since urban green development data are not directly available, we construct an indicator evaluation system from three dimensions: socioeconomic, quality of life and ecological environment, based on the research literature, and use the entropy value method to measure green development data^32–34^, with the following calculation steps (Table 1).

- Data standardisation
  The aim of standardising the data is to remove the effects of dimensionality, thus making the data more comparable. In addition, data standardisation can make the data homogenous so that indicators of different natures have the same direction of action on the evaluation results. We use the extreme difference standardisation method for dimensionless processing with the following formula.
  $$ {\text{Positive indicators}}:\;\;x_{ij}^{\prime } = \frac{{x_{ij} - Min(x_{j} )}}{{Max(x_{j} ) - Min(x_{j} )}} $$
  $$ {\text{Negative indicators}}:\;\;x_{ij}^{\prime } = \frac{{Max(x_{j} ) - x_{ij} }}{{Max(x_{j} ) - Min(x_{j} )}} $$
  where $i$ and $j$ are prefectures and indicators, respectively $x_{ij}$ and $x_{ij}^{\prime }$ denote raw data and extreme deviation normalised data, respectively, and $Min(x_{j} )$ and $Max(x_{j} )$ denote the minimum and maximum values of raw data, respectively.
- Calculating information entropy $e_{j}$
  A sample of $n$ prefecture-level cities and $m$ evaluation indicators is assumed, and the weighting of the characteristics of prefecture-level city $i$ under indicator $j$ is shown below.
  $$ P_{ij} = \frac{{x_{ij}^{\prime } }}{{\sum\nolimits_{i = 1}^{n} {x_{ij} } }} \, (i = 1,2,3,...,n;j = 1,2,3,...,m) $$
  The information entropy value is calculated for indicator $j$.
  $$ e_{j} = - \frac{1}{\ln n}\sum\limits_{i = 1}^{n} {P_{ij} } \ln P_{ij} ,0 \le e_{j} \le 1 \, (j = 1,2,3,...,m) $$
- Calculation of weights and overall score
  The weight $w_{j}$ of the j-th index is calculated and the comprehensive score $Score_{i}$ of green development of prefecture-level city $i$ is determined.
  $$ w_{j} = \frac{{1 - e_{j} }}{{\sum\nolimits_{j = 1}^{m} {(1 - e_{j} )} }} $$
  $$ Score_{i} = \sum\limits_{i = 1}^{n} {w_{j} x_{ij} } \, (i = 1,2,3,...,m;\;\; j = 1,2,3,...,n) $$
  As the coefficient is too small due to the small indicator of the independent variable, we treat measurement A by multiplying it by 10 (Table 1).

**Table 1:** Urban green development evaluation index system.

First-level indicators	Second-level indicators	Measurement indicators	Attribute
Socioeconomic	Economic development	Regional GDP	+
	Resident income	Per capita disposable income	+
	Government investment	Fixed asset investment	+
	Income distribution	Gini coefficient	−
Quality of life	Cultural facilities	Library collections per capita	+
	Convenient transportation	Road area per capita	+
	Internet popularity	Internet penetration	+
	Employment stability	Unemployment rate	−
Ecological environment	Atmospheric pollution	Emissions per unit of exhaust gas	−
	Water contamination	Unit effluent discharge	−
	Greenhouse gas	Emissions per unit of CO_2_	−
	Environmental regulation	Industrial pollution control intensity	+
	Urban greenery	Green space per capita	+

*Core independent variable* The core explanatory variable in this paper is the smart city pilot policy and is assigned a dummy variable in the form of a location dummy of 1 if the city is on the smart city list and 0 otherwise and a time dummy of 1 if the time is after the implementation of the smart city pilot policy and 0 otherwise, with the interaction term representing the policy variable for smart city construction, denoted as Smartcity. The coefficient of the interaction term is the estimated value that is the focus of this paper; if it is significantly positive, then the smart city pilot policy can significantly contribute to urban green development.

*Control variables* Based on a review of the literature, the control variables selected for this paper are as follows: (1) urbanisation rate, measured as the ratio of the urban population to the total population, denoted as town; (2) level of financial development, measured as the ratio of total loans of all balances of financial institutions to GDP at the end of the year, denoted as fin; (3) investment in scientific research, measured as expenditure on science and technology, denoted as rd; (4) level of education of the population, measured as expenditure on education, denoted as edu; (5) the degree of government intervention, measured using public budget expenditure in municipalities as a percentage of GDP, denoted govern; and (6) population size, measured using year-end population numbers, denoted peop.

*Intermediate variables* In this paper, industrial structure and green technological innovation are selected as mediating variables for analysis. Referring to Ye et al., the industrial structure is measured by the share of the secondary industry in GDP^35^. Green technological innovation is measured by adding one to the logarithm of green patent applications in prefecture-level cities and then analysing the mechanism of action involved in the impact of smart city construction on urban green development (Table 2).

**Table 2:** Variable definitions and descriptions.

Variable properties	Variable name	Abbreviations	Measurement methods
Dependent variable	Urban green development	*UGD*	Calculated by entropy method
Core independent variable	Interaction term	*Smartcity*	–
Control variables	Urbanisation rate	*town*	Urbanisation rate = number of urban population/total population
	Financial development level	*fin*	Financial development level = total balance loans/GDP
	Research and development	ln*rd*	Logarithm of science and technology expenditure
	Education level of the population	ln*edu*	Logarithm of education expenditure
	Level of government intervention	*govern*	Level of government intervention = public budget expenditure/GDP
	Population size	ln*peop*	Logarithm of year-end population
Intermediate variables	Industrial structure	*indus*	Industrial structure = secondary GDP/total GDP
	Green technology innovation	ln*gti*	Logarithm of the number of green patent applications in prefecture-level cities plus one

### Data use

**Figure 2:**
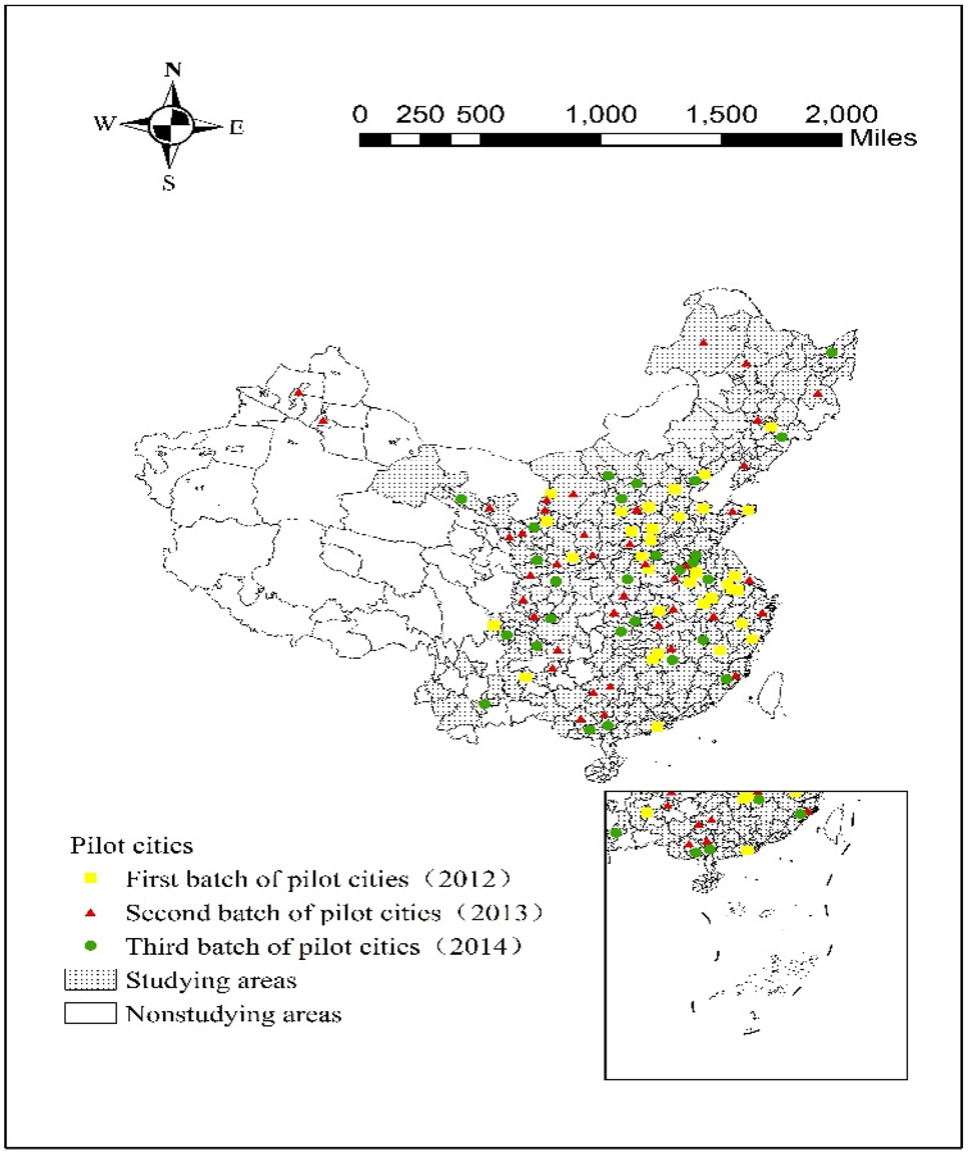
Sample distribution.

China officially promoted the construction of smart cities in 2012, and the second and third batches of smart city pilots were announced in 2013 and 2014, respectively. This paper treats the smart city pilot policy as a quasinatural experiment and uses the asymptotic DID method to analyse the impact of smart city construction on urban green development (Fig. 2).

As the pilot list includes counties and urban areas, the following treatment is performed when selecting the empirical sample: to avoid underestimating the impact of the smart city pilot policy on urban green development and to make the regression results more robust, the prefecture-level cities where the counties and urban areas are located in the list are excluded from the study sample, and only prefecture-level municipal units are retained in the final sample (Table 3).

**Table 3:** Sample distribution.

Treatment group	Comparison group
Hebei(5) Shanxi(6) Inner Mongolia(4) Liaoning(1) Jilin(3) Heilongjiang(3) Jiangsu(6) Zhejiang(3) Anhui(10) Fujian(3) Jiangxi(4) Shandong(4) Henan(6) Hubei(6) Hunan(2) Guangdong(1) Guangxi(6) Sichuan(6) Yunnan(1) Guizhou(3) Shaanxi(5) Gansu(6) Ningxia(4) Xinjiang(2)	Hebei(5) Shanxi(3) Inner Mongolia(4) Liaoning(11) Jilin(2) Heilongjiang(6) Jiangsu(1) Zhejiang(5) Anhui(3) Fujian(4) Jiangxi(2) Shandong(5) Henan(8) Hubei(6) Hunan(5) Guangdong(13) Guangxi(8) Hainan(2) Sichuan(10) Yunnan(6) Shaanxi(5) Gansu(5) Qinghai(1) Ningxia(1)

Figures in brackets indicate the number of prefecture-level city samples in the treatment and comparison groups.

Based on the above procedure, this paper uses a panel of 221 prefecture-level cities in China from 2006 to 2020 to analyse the possible impact of smart city pilot policies on urban green development. The analysis in this paper does not consider Tibet or Hong Kong, Macao or Taiwan due to serious data deficiencies in these regions. While processing the data, data from relevant autonomous prefectures, county-level cities and urban areas are excluded, and only the city data are retained for analysis. A small amount of missing data is interpolated using the linear interpolation method. Data for the study were obtained from the wind database, the China Urban Statistics Yearbook and data publicly available from provincial statistical bureaus (Table 4).

**Table 4:** Descriptive statistics.

Variables	Full sample			Comparison group			Treatment group		
	N obs	Mean	SD	N obs	Mean	SD	N obs	Mean	SD
*UGD*	3315	0.8087	0.4824	1815	0.7393	0.4249	1500	0.8927	0.5320
*Smartcity*	3315	0.2431	0.4290	1815	0.0000	0.0000	1500	0.5373	0.4988
*town*	3315	0.5018	0.1516	1815	0.4775	0.1458	1500	0.5312	0.1532
*fin*	3315	1.9915	1.4613	1815	1.9612	1.3378	1500	2.0282	1.5978
ln*rd*	3315	9.5863	1.4537	1815	9.3331	1.3057	1500	9.8926	1.5613
ln*edu*	3315	11.4639	1.0202	1815	11.3071	0.9447	1500	11.6537	1.0748
*govern*	3315	0.1952	0.1170	1815	0.2125	0.1316	1500	0.1743	0.0923
ln*peop*	3315	5.7699	0.6825	1815	5.7416	0.6621	1500	5.8042	0.7052
*indus*	3315	0.4713	0.1224	1815	0.4555	0.1134	1500	0.4903	0.1300
ln*gti*	3315	2.0741	1.5399	1815	1.7520	1.3688	1500	2.4638	1.6423

### Model setting

To analyse the impact of smart city construction on urban green development, this paper adopts a progressive DID method to treat smart city construction as a quasinatural experiment to analyse its treatment effects on urban green development. This paper further constructs a model to assess whether smart city construction has promoted urban green development by comparing the differences between pilot areas and nonpilot areas. The econometric model constructed in this paper is as follows.

$$ UGD_{it} = \beta_{0} + \beta_{1} Smartcity_{it} + X_{it}^{\prime } \beta_{2} + \mu_{i} + \delta_{t} + \varepsilon_{it} $$

Of these, UGD_it_ is the city green development index for city i at time t; Smartcity_it_ is the policy variable in the DID method, the interaction term mentioned above, which represents smart city construction; X_it_ is the control variable mentioned above; $\mu_{i}$ is the time fixed effect; $\delta_{t}$ is the location fixed effect; and $\varepsilon_{it}$ is the random error term. $\beta_{1}$ is the coefficient estimate that is the focus of this paper, for which a significant positive value indicates that smart city construction can significantly contribute to urban green development.

## Empirical analysis

### DID results analysis

The use of the DID method presupposes that the treatment and comparison groups should have the same trend of change before the policy shock, i.e., they should pass the parallel trend test. The parallel trend test conducted in this paper demonstrates the applicability of the double difference method in this study; the parallel trend test is shown in the robustness test below. Table 5 shows the baseline regression for this paper, and columns (1)–(7) show the regression results after the control variables are gradually added. The estimated coefficient of the policy variable Smartcity_it_ is still significantly positive at the 1% level after the control variables are gradually added, indicating that smart city construction significantly promotes urban green development. Hypothesis 1 is argued in this paper.

**Table 5:** DID Results analysis.

Variables	Dependent variable: UGD						
	(1)	(2)	(3)	(4)	(5)	(6)	(7)
*Smartcity*	0.1162*** (0.0100)	0.1138*** (0.0100)	0.1094*** (0.0099)	0.0984*** (0.0099)	0.0984*** (0.0099)	0.0937*** (0.0098)	0.0933*** (0.0098)
*town*		0.3279*** (0.0908)	0.2735*** (0.0895)	0.2326** (0.0911)	0.2366*** (0.0914)	0.2158** (0.0903)	0.2369*** (0.0916)
*fin*			− 0.0414*** (0.0059)	− 0.0325*** (0.0059)	− 0.0329*** (0.0060)	− 0.0243*** (0.0060)	− 0.0238*** (0.0060)
ln*rd*				0.0425*** (0.0063)	0.0433*** (0.0065)	0.0447*** (0.0067)	0.0429*** (0.0067)
ln*edu*					− 0.0061 (0.0089)	0.0039 (0.0094)	0.0014 (0.0094)
*govern*						− 0.3649*** (0.0845)	− 0.3554*** (0.0844)
ln*peop*							0.0908 (0.0552)
Constant	0.7804*** (0.0035)	0.6164*** (0.0456)	0.7273*** (0.0486)	0.3256*** (0.0711)	0.3862*** (0.1095)	0.3243*** (0.1202)	− 0.1673 (0.3257)
City FE	Yes	Yes	Yes	Yes	Yes	Yes	Yes
Year FE	Yes	Yes	Yes	Yes	Yes	Yes	Yes
Observations	3315	3315	3315	3315	3315	3315	3315
R-squared	0.915	0.915	0.918	0.920	0.920	0.921	0.921

Robust standard errors in parentheses. ***p < 0.01, **p < 0.05, *p < 0.1.

Looking further at the regression coefficients of the control variables in this paper, the urbanisation rate and investment in research significantly contribute to green urban development, while financial development and the degree of government intervention have negative impacts on green urban development. The coefficient of the urbanisation rate (town) is significantly positive because smart city construction represents an advanced stage of China's new urbanisation process. The manner in which the ecological environment is emphasised in new urbanisation is different from that in traditional urbanisation, which develops the economy at the expense of the environment. The reason why scientific research investment (lnrd) plays a significant role in promoting urban green development is that scientific research investment promotes technological innovation in smart city construction and policy formulation, which is conducive to improving the efficiency of energy use, optimising the allocation of resources, and providing decision support for the green attributes of smart city construction. The estimated coefficient of the degree of government intervention (govern) is significantly negative because the government's public budget expenditure is aimed at safeguarding and improving people's livelihood and promoting the country's economic and social development. Along with infrastructure improvements and steadily developing economic levels, environmental problems are becoming increasingly serious, thus hindering urban green development to some extent. The reason for the significantly negative estimated coefficient on the level of financial development (fin) is that financial institutions are more inclined to focus on short-term returns on investment, whereas the green development of smart cities tends to require longer investment cycles and slower rates of return. Therefore, financial institutions may be reluctant to invest in long-term, potentially less rewarding green projects for smart city construction if they are overly concerned with short-term returns. In addition, the implementation and effectiveness of green projects in smart cities may be difficult for financial institutions to accurately assess due to information asymmetry, which in turn may curb investment willingness.

### Robustness tests

#### Parallel trend test

**Figure 3:**
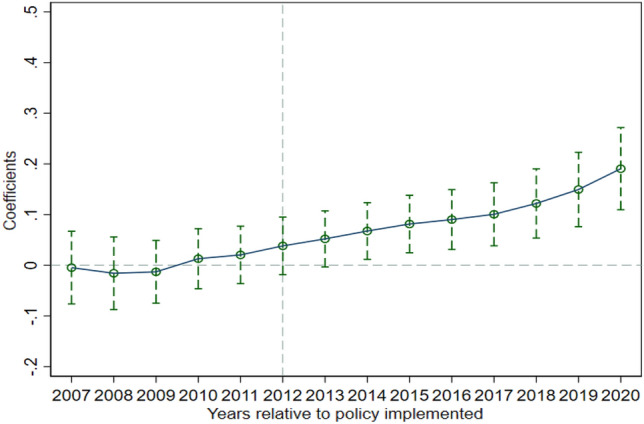
Parallel trend test.

The core assumption of the DID model is that the treatment and comparison groups should have the same trend of change before the policy is implemented. The parallel trend test model constructed in this paper is shown below.

$$ UGD_{it} = \alpha + \sum\limits_{k \ge - 5}^{8} {\beta_{k} D_{it}^{k} } + \sum {\lambda_{j} } X_{it} + \phi T_{jt} + \mu_{i} + \delta_{t} + \varepsilon_{it} $$

where $D_{it}^{k}$ is the dummy variable for the current period of smart city construction, k < 0 is the year before smart city construction, k > 0 is the year after smart city construction, T_jt_ is the time trend variable, and the remaining variables are consistent with the previous expression. Figure 3 shows the parallel trend test graph. Before smart city construction began, the treatment group and the control group have a consistent trend of change; in 2012 for the current period of smart city construction and in 2013, urban green development has no significant impact, indicating that the policy has a time lag. After smart city construction, a long-term positive effect is found. Therefore, smart city construction can significantly promote urban green development, with a long-term effect.

#### Placebo test

**Figure 4:**
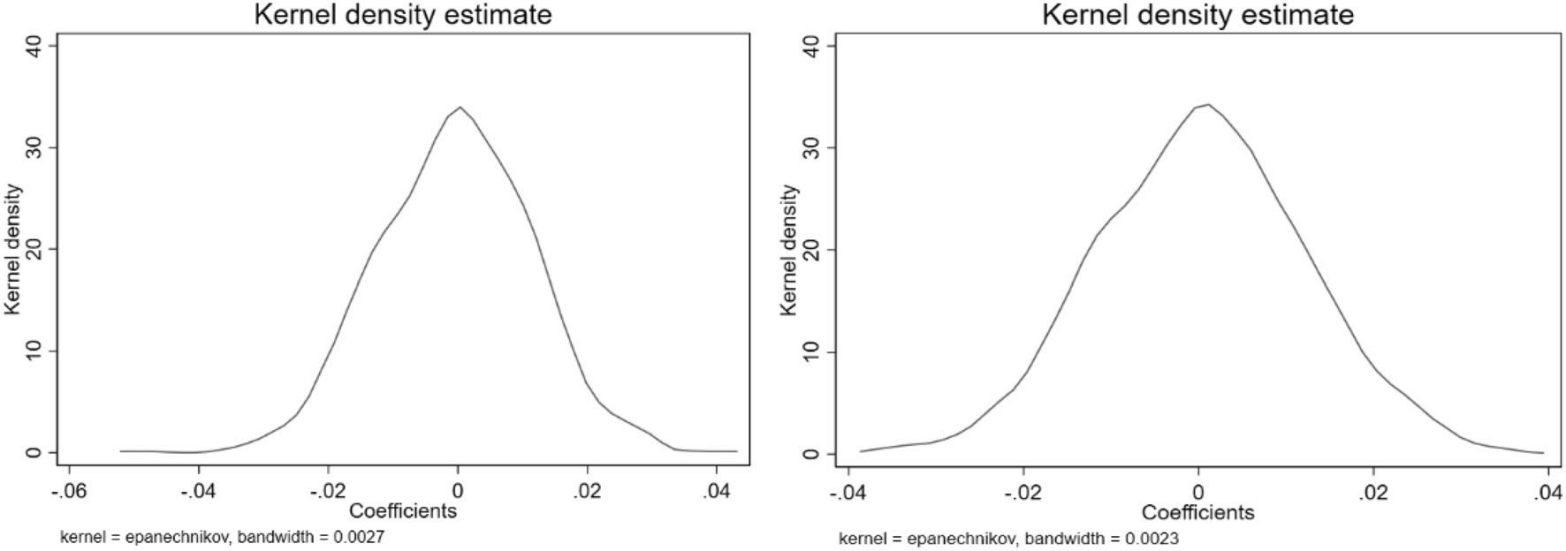
Placebo test.

Smart city construction may be influenced by unobservable factors; hence, this paper refers to Li et al. to conduct random sampling to ensure that the impact of smart city construction on a particular city is randomised, which eliminates the influence of unobservable factors^36^. The coefficient estimates of Smartcity_it_ are as follows.

$$ \hat{\beta } = \beta + \theta \times \frac{{{\text{cov}} (Smartcity_{it} ,\varepsilon_{it} \left| {X_{it} } \right.)}}{{{\text{var}} (Smartcity_{it} \left| {X_{it} } \right.)}} $$

where X_it_ is the control variable mentioned above and $\theta$ is an unobservable factor. If $\hat{\beta }$ is to be an unbiased estimator to avoid inconsistent estimates, then parameter $\theta = 0$ should be made. However, direct testing of parameter $\theta$ is less tractable; thus, random sampling is performed to generate smart city construction cities to make parameter $\beta = 0$. If $\hat{\beta } = 0$ exists, then parameter $\theta = 0$ can be introduced^37^. Figure 4 shows the kernel density plots of the coefficient estimates obtained after 1000 (left panel) and 2000 (right panel) random samplings. The $\hat{\beta }_{random}$ coefficient estimates are concentrated at approximately 0 and approximately obey a normal distribution after random sampling is conducted; thus, the parameter $\theta = 0$ can be inferred, indicating that the findings of this paper are robust.

#### Nonrandom selection impact test

Although the use of the DID method to evaluate the impact of smart city construction on urban green development can, to a certain extent, avoid the unobservable endogenous factors that belong to the "common trend" between the treatment and control groups, smart city construction is policy-oriented, and the list of construction areas is not randomly generated. Based on this situation, the interaction term of urban factors and time trends is introduced and the following econometric model is constructed.

$$ UGD_{it} = \beta_{0} + \beta_{1} Smartcity_{it} + C_{i} \times Tim_{t} + X_{it}^{\prime } \beta_{2} + \mu_{i} + \delta_{t} + \varepsilon_{it} $$

where C_i_ denotes the city attribute factor, C_i_ includes provincial capitals, subprovincial cities and areas east of the Hu Huanyong line, Tim_t_ is the time trend variable, and the remaining variables are the same as in the previous statement. As shown in Table 6, the interaction term Smartcity_it_ coefficient is still significantly positive after controlling for the city attribute factors, and the conclusions of this paper are robust.

**Table 6:** Nonrandom selection effects test.

Variables	Dependent variable: UGD				
	(1) Nonrandom factors	(2) Radius	(3) Nearest	(4) Kernel	(5) llr
*Smartcity*	0.1038*** (0.0265)	0.0248*** (0.0074)	0.0358*** (0.0115)	0.0310*** (0.0054)	0.0314*** (0.0053)
Provincial capitals $\times Tim$	0.2041*** (0.0368)				
Subprovincial cities $\times Tim$	0.5870*** (0.0683)				
East of Hu Huanyong Line $\times Tim$	− 0.0495* (0.0269)				
Constant	− 0.1056 (0.3072)	− 1.8266*** (0.2556)	− 1.3914*** (0.3578)	− 1.7944*** (0.2060)	− 1.8868*** (0.2069)
Control	Yes	Yes	Yes	Yes	Yes
City FE	Yes	Yes	Yes	Yes	Yes
Year FE	Yes	Yes	Yes	Yes	Yes
Observations	3315	3299	3301	3301	3301
R-squared	0.927	0.915	0.820	0.949	0.956

Robust standard errors in parentheses. ***p < 0.01, **p < 0.05, *p < 0.1.

**Figure 5:**
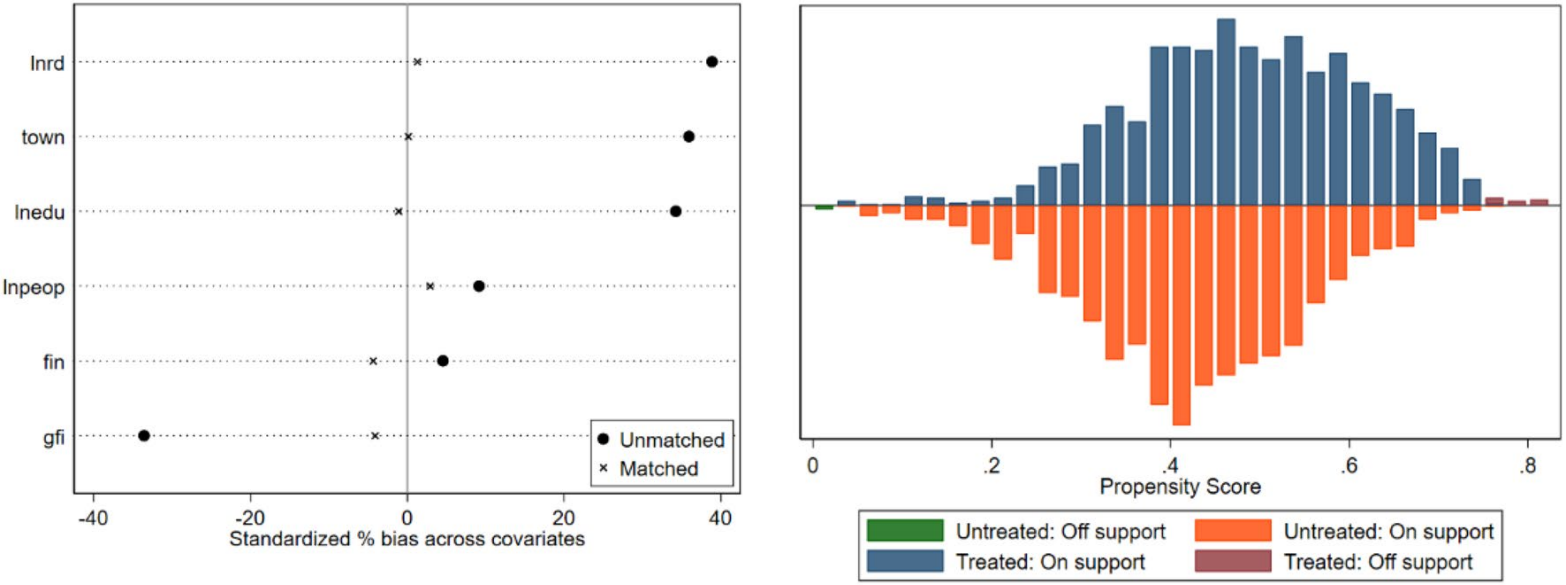
Propensity score matching balance test.

In addition, to further demonstrate the robustness of the regression results, a combination of PSM and the DID method is adopted to analyse the effect of smart city construction on urban green development, which can overcome the effect of selection bias. In this paper, three matching methods, kernel matching, radius matching and local linear regression matching, were used to perform PSM-DID estimation. Figure 5 shows the results of the equilibrium test after kernel matching, which shows that the standardised deviations of the covariates are reduced and concentrated to approximately 0 after matching (Fig. 5 left) and that the samples of the treatment and control groups mostly lie within the common range of values (Fig. 5 right), which is a good matching result. All other matching methods passed the balance test.

Columns (2)–(5) in Table 6 show the regression results after radius matching, nearest neighbour matching, kernel matching and local linear regression matching. The coefficients of the policy variables are still significantly positive at the 1% level, indicating that the effect of smart city construction on urban green development is not affected by selection bias and that the regression results in this paper are still robust.

#### Heterogeneity analysis

As different locations of cities have different economic development, Chinese prefecture-level cities are divided into three subsamples, namely, eastern, central and western, based on their geographical locations and then separate regressions for geographical location heterogeneity analysis are performed. As shown in Table 7, smart city construction has a significant impact on urban green development in the eastern, central and western regions of China, with the greatest impact on urban green development in the eastern region, followed by the central region and finally by the western region. The reason for this is that eastern China has a better foundation in high-tech industries and is gradually transforming and upgrading to smart manufacturing, the internet, artificial intelligence and other fields, which provides strong technological support for promoting the construction of smart cities. For example, the application of big data, the Internet of Things, artificial intelligence and other technologies can not only optimise city operations and improve efficiency but also promote industrial transformation and upgrading and green development. On the other hand, after a long period of high-intensity industrialisation and urbanisation, the ecological environment in eastern China is under greater pressure. The construction of smart cities can effectively prevent and repair ecological problems with information technology. In the new infrastructure of big data, Internet of Things, cloud computing, etc., the new infrastructure of the eastern part of the country is growing in parallel with the urban scale and industrial structure of the eastern part of the country, forming a coupling state, thus maximising the policy effect of smart city construction on urban green development.

**Table 7:** Heterogeneity analysis.

Variables	Dependent variable: UGD		
	(1) Eastern	(2) Central	(3) Western
*Smartcity*	0.1457*** (0.0167)	0.0754*** (0.0141)	0.0737*** (0.0141)
Constant	− 2.7974*** (0.8030)	0.3389 (0.3915)	0.9205 (0.6801)
Control	Yes	Yes	Yes
City FE	Yes	Yes	Yes
Year FE	Yes	Yes	Yes
Observations	1035	1140	1140
R-squared	0.942	0.908	0.915

Robust standard errors in parentheses. ***p < 0.01, **p < 0.05, *p < 0.1.

#### Endogeneity issues

The nonrandom nature of smart city construction may lead to endogeneity problems when analysing the impact of smart city construction on urban green development. Therefore, in this paper, instrumental variable estimation (IV) is used to address possible endogeneity problems. Topographic relief (rdls) is chosen as the instrumental variable for the core explanatory variable Smartcity_it_. On the one hand, topographic relief is closely related to the location of smart city construction, and the relevance assumption of the instrumental variable is satisfied; on the other hand, topographic relief, as geographical data, does not affect urban green development, and the exogeneity assumption of the instrumental variable is satisfied^38^. Furthermore, as topographic relief data do not change over time and have research limitations, the interaction term T-rdls is introduced between the instrumental variables and each year's time dummy variable and is then used as the instrumental variable for the empirical analysis to measure changes in the time dimension. The instrumental variable model is constructed as shown below.

$$ \begin{aligned} UGD_{it} & = \xi_{0} + \xi_{1} S{\text{martcity}}_{it} + \sum {\xi_{j} X_{it} + \varepsilon_{it} } \hfill \\ Smartcity_{it} & = \xi_{0}^{1} + \xi_{1}^{1} T - rdls_{it} + \sum {\xi_{j}^{1} } X_{it} + \mu_{i} + \delta_{t} + \varepsilon_{it}^{1} \hfill \\ UGD_{it} & = \xi_{0}^{2} + \xi_{1}^{2} Smartcity_{it} - hat + \sum {\xi_{j}^{2} X_{it} + \mu_{i} + \delta_{t} + \varepsilon }_{it}^{2} \hfill \\ \end{aligned} $$

where parameters $\xi_{1}$, $\xi_{1}^{1}$ and $\xi_{1}^{2}$ denote the ordinary least square estimates, IV estimates the regression coefficients for the first and second stages, and the remaining variables are the same as above.

Column (1) of Table 8 shows the results of the one-stage regression, where the estimated coefficients of the interaction term T-rdls for the topographic relief and time dummy variables are significantly positive. Column (2) shows the results of the two-stage regression, where the regression coefficient of the interaction term Smartcity_it_ is smaller than the case of the previous benchmark regression, indicating that the impact of smart city construction on urban green development will be overestimated if the endogeneity issue is not accounted for.

**Table 8:** Instrumental variable estimation.

Variables	Instrumental variable: T-rdls	
	(1)	(2)
*Smartcity*		0.0648*** (0.0224)
*T-RDLS*	0.4866*** (0.0161)	
Constant	− 1.1616*** (0.0729)	− 2.2750*** (0.0881)
Control	Yes	Yes
City FE	Yes	Yes
Year FE	Yes	Yes
Underidentification test		446.556 [0.000]
Weak identification test		2082.340 {16.38}
Observations	3315	3315
R-squared	0.516	0.905

Robust standard errors in parentheses. ***p < 0.01, **p < 0.05, *p < 0.1;The Kleibergen‒Paap rk LM statistic was used for the underidentification test, and the numbers in [] are their p values; the weak identification test refers to the Donald Wald-F statistic, and the Stock-Yogo test 10% level thresholds are in {}.

## Further analysis

### Mechanism analysis

In this paper, to further explore the possible mechanisms of the impact of smart city construction on urban green development, green technology innovation is measured by adding one to the number of green patent applications in prefecture-level cities and taking the proportion of secondary industry in GDP as the measurement variable of industrial structure. The mechanism effect model is shown below.

$$ \ln gti_{it} = \alpha_{0} + \alpha_{1} Smartcity_{it} + X_{it}^{\prime } \alpha_{2} + \mu_{i} + \delta_{t} + \varepsilon_{it} $$

$$ UGD_{it} = \gamma_{0} + \gamma_{1} Smartcity_{it} + \gamma_{2} \ln gti_{it} + X_{it}^{\prime } \gamma_{3} + \mu_{i} + \delta_{t} + \varepsilon_{it} $$

$$indus_{it} = \varphi_{0} + \varphi_{1} Smartcity_{it} + X_{it}^{\prime } \varphi_{2} + \mu_{i} + \delta_{t} + \varepsilon_{it}$$

$$ UGD_{it} = \phi_{0} + \phi_{1} Smartcity_{it} + \phi_{2} indus_{it} + X_{it}^{\prime } \phi_{3} + \mu_{i} + \delta_{t} + \varepsilon_{it} $$

Column (1) of Table 9 shows the impact of smart city construction on green technology innovation, and the findings show that smart city construction significantly promotes green technology innovation. Column (2) shows that the coefficients of policy variables and green technology innovation are both significant at the 1% level, indicating that green technology innovation is an important channel through which smart city construction affects urban green development and that smart city construction significantly contributes to urban green development through green technology innovation. Column (3) shows the impact of smart city construction on industrial structure, and the empirical results show that smart city construction has a negative impact on industrial structure, which is the same as theoretical expectations. Column (4) shows that the coefficients of both industrial structure and policy variables are significant, indicating that industrial structure is an important channel through which smart city construction affects urban green development and that smart city construction significantly contributes to urban green development through industrial structure. Hypothesis 2 of this paper is argued.

**Table 9:** Mechanism analysis.

Variables	(1)	(2)	(3)	(4)
	Lngti	UGD	indus	UGD
*Smartcity*	0.2286*** (0.0362)	0.0866*** (0.0098)	− 0.0148*** (0.0031)	0.0913*** (0.0101)
ln*gti*		0.0291*** (0.0044)		
*indus*				− 0.1306** (0.0583)
Constant	− 9.9744*** (1.2226)	0.1224 (0.3256)	0.4136*** (0.0925)	− 0.1133 (0.3317)
Control	Yes	Yes	Yes	Yes
City FE	Yes	Yes	Yes	Yes
Year FE	Yes	Yes	Yes	Yes
Observations	3315	3315	3315	3315
R-squared	0.888	0.922	0.872	0.921

Robust standard errors in parentheses. ***p < 0.01, **p < 0.05, *p < 0.1.

### Spatial econometric analysis

In the above section, the causal relationship between smart city construction and urban green development is identified using the progressive DID model, but the spatial factors of smart city construction affecting urban green development are not considered. In this section, a DID spatial Durbin model (SDMDID) is established to introduce and decompose these spatial factors according to how their impact on smart city construction influences urban green development; this process can analyse the urban green development spillover effect of smart city construction. The model is set as follows.

$$ \begin{aligned} UGD_{it} & = \beta_{0} + \rho W \times UGD_{it} + \beta_{1} Smartcity_{it} + \beta_{2} X_{it}^{\prime } + \gamma_{1} W \times Smartcity_{it} \hfill \\ & \quad  + \gamma_{2} W \times X_{it}^{\prime } + \mu_{i} + \delta_{t} + \varepsilon_{it} \hfill \\ \end{aligned} $$

where W is the spatial weight matrix, the binary adjacency matrix is selected as the spatial weight matrix in this paper, $W \times UGD_{it}$ denotes the spatial lag term of urban green development, $W \times Smartcity_{it}$ denotes the spatial lag term of smart city construction, $W \times X_{it}^{\prime }$ denotes the spatial lag term of the control variables, and the remaining variables are the same as in the previous expressions.

#### Global spatial correlation test

The premise of using the SDMDID model is that the explanatory variables are spatially correlated, i.e., Moran’s I should be significantly nonzero. This paper uses Moran’s I to test the spatial correlation between smart city construction and urban green development. Table 10 shows that the Moran’s I values of both urban green development and smart city construction are greater than zero, indicating that both are spatially correlated, i.e., the implementation of smart city construction will not only have an impact on the implementation of smart city construction and affect urban green development in this region but also affect other adjacent regions.

**Table 10:** Global spatial correlation test.

Year	Urban green development	Smart city construction
	Moran's I	Moran's I
2012	0.195	0.643
2013	0.206	0.736
2014	0.212	0.806
2015	0.212	0.806
2016	0.201	0.806
2017	0.162	0.806
2018	0.155	0.806
2019	0.145	0.806
2020	0.144	0.806

#### Spatial econometric model analysis

The paper further examines and analyses whether the DID spatial Durbin model degenerates into either a DID spatial lag model or a DID spatial error model. Table 11 illustrates that both the LR test and the Wald test significantly reject the original hypothesis, fully demonstrating the applicability of the spatial Durbin model in this study. Based on the results of the Hausman test, time and space double fixed effects are chosen to analyse the spillover effects of urban green development of smart city construction.

**Table 11:** SDMDID model applicability test.

Type of tests	Statistical values
LR_spatial_ lag	42.32***
LR_spatial_ error	42.24***
Wald_spatial_lag	59.24***
Wald_spatial_error	17.03***
Hausman test	428.97***

Column (1) of Table 12 shows the overall regression results, while columns (2)-(4) show the results after decomposition by partial differencing, indicating the direct effect, the spatial spillover effect and the total effect, respectively. The direct effect indicates that smart city construction has a significant contribution to urban green development, which is consistent with the findings from the previous baseline regression. The spatial spillover effect indicates that the implementation of smart city construction will benefit urban green development in neighbouring cities. The implementation of smart city construction will cause neighbouring cities to change their industrial structure, eliminate inefficient production capacity and improve energy efficiency through imitation and learning, thus promoting urban green development in neighbouring cities. Therefore, smart city construction has a good spatial radiation effect on neighbouring areas.

**Table 12:** Estimation of spatial measures and decomposition of spatial effects.

Variables	Dependent variable: UGD			
	(1) Main	(2) LR_Direct	(3) LR_Indirect	(4) LR_Total
*Smartcity*	0.1240*** (0.0102)	0.1294*** (0.0110)	0.0461* (0.0266)	0.1755** (0.0318)
rho	0.5505*** (0.0139)	–	–	–
sigma2_e	0.0244*** (0.0006)	–	–	–
Control	Yes	Yes	Yes	Yes
City FE	Yes	Yes	Yes	Yes
Year FE	Yes	Yes	Yes	Yes
Observations	3315	3315	3315	3315
R-squared	0.221	0.221	0.221	0.221
Number of id	221	221	221	221

Standard errors in parentheses. ***p < 0.01, **p < 0.05, *p < 0.1.

## Conclusions

Smart city construction has given rise to the emergence and concentration of new industries and injected new momentum into the development of the digital economy. With the in-depth development of the digital economy and information technology, smart city construction has gradually become an important trend in urban development. Therefore, this paper uses the panel data of 221 prefecture-level cities in China from 2006 to 2020 to employ the asymptotic double difference method to empirically analyse whether smart city construction will promote urban green development The following conclusions are drawn. First, smart city construction significantly promotes urban green development and has a long-term promotion effect. Second, an analysis of intermediary effects shows that urban industrial structure and technological innovation are important channels through which smart city construction affects urban green development. Third, urban green development in neighbouring areas is promoted by the positive spatial spillover effect of smart city construction. In summary, this paper proposes corresponding policy recommendations at three levels: macro, meso and micro:

First, the government should actively expand the scale of smart city construction, expand the scope of smart city pilots, further promote the development process of smart cities, and further introduce relevant policies to support the construction of smart cities. In promoting the construction of smart cities, the use of “one city, one policy” should be tailored to local conditions to create a smart city program. For example, the eastern region of China benefits the most from policy; hence, smart city construction can be prioritised in the eastern region as a way to promote urban green development to a greater extent. At the same time, governments worldwide should also deepen cooperation and abandon the “fragmented, beggar-thy-neighbour” development model. Regional governments of smart city construction should promote their construction experience to support the digitalised intelligent construction of neighbouring cities, deepen cobusiness, coconstruction and sharing among resource elements, actively expand the positive spatial spillover effect brought by smart city construction, and jointly promote urban green development.

Second, major industries need to fully embrace the policy dividend and promote digital industrialisation and industrial digitisation. Traditional resource-intensive and labour-intensive industries must take full advantage of the smart economy and transformation opportunities to fully benefit from the industrial structure effect, thus creating more ecological and social benefits while promoting industrial upgrading. These actions will promote regional green development.

Third, enterprises, universities and research institutes should continue to develop intelligence, digitalisation and informatization, improve research and development of pollution prevention technology and further promote the overflow of green technology and knowledge. They should encourage other enterprises to follow their example via the catfish effect and competition effect. Moreover, they should continue to support the spatial spillover effect, which allows the impact of green development to radiate to neighbouring areas, thus increasing urban green development.
